# Concurrent Middle Ear and Vertebral Tuberculosis Secondary to *Mycobacterium Africanum* in an Immunocompetent Young Adult in a Low‐Incidence Setting: Diagnostic Value of Middle Ear Biopsy

**DOI:** 10.1155/crdi/5812867

**Published:** 2026-10-16

**Authors:** Sabirah N. Kasule, George S. Zacharia, Abelard C. Desrosiers, Shalini Penikilapate, Marcos A. Molina, Srinivasan Krishna, Cosmina B. Zeana

**Affiliations:** ^1^ Division of Infectious Diseases Department of Internal Medicine BronxCare Health System Bronx New York USA bronxcare.org; ^2^ Internal Medicine Residency Department of Internal Medicine BronxCare Health System Affiliated With Icahn School of Medicine at Mt Sinai Bronx New York USA; ^3^ School of Medicine The City University of New York (Cuny) New York New York USA; ^4^ Department of Otolaryngology BronxCare Health System Bronx New York USA bronxcare.org

**Keywords:** cervical spine, extrapulmonary tuberculosis, New York, otic

## Abstract

Otic tuberculosis is an exceedingly rare manifestation of extrapulmonary tuberculosis (EPTB) and typically mimics more common otologic conditions, leading to delayed diagnosis and treatment. This report discusses a case of middle ear tuberculosis in a New York City–born‐and‐raised young woman. She presented with ear drainage, hearing loss, generalized weakness, and a painful lump on her posterior neck. She was subsequently diagnosed with multiple abscesses along the cervical and thoracic spine and a large mass in her middle ear that protruded through her tympanic membrane into the external auditory canal. Biopsy of the protruding part of the ear mass revealed non‐necrotizing granulomatous inflammation and acid‐fast bacilli on pathology. *Mycobacterium tuberculosis* (MTB) complex PCR was positive, and mycobacterial cultures grew *Mycobacterium africanum*. She was promptly initiated on rifampin, isoniazid, pyrazinamide, and ethambutol (RIPE) with clinical improvement of her symptoms.

## 1. Introduction

In 2023, tuberculosis (TB) returned to being the world’s leading cause of death from a single infectious agent after 3 years of being upstaged by the COVID‐19 pandemic. According to the WHO, 10.8 million were diagnosed with TB in 2023, and 1.25 million succumbed to the disease [1]. Pulmonary TB is the most well‐known form of TB, but extrapulmonary tuberculosis (EPTB) cases are on the rise and accounted for 16% of the 7.1 million new and relapsed incident cases of TB in 2019 [2]. This represents an increase from 15% of 7 million in 2018 [3] and 14% of 6.4 million in 2017 [4]. Nevertheless, EPTB still presents a diagnostic challenge for clinicians as affected organ systems, which can include the eyes or the brain, are difficult to obtain samples from, and collected samples are often paucibacillary [5]. Tuberculous infection of the spine, or Pott’s disease, and otic TB are especially rare forms of EPTB. Simultaneous infection is even more unusual, and most reported cases in the literature appear to arise from hematogenous spread in the setting of reactivation and dissemination rather than direct extension from the ear to the spine or vice versa [6, 7]. Here, we present a case of concurrent otic and spinal TB in a young woman with no appreciable risk factors who was living in a country with low incidence of TB. The diagnosis was made by biopsy of her ear lesion. Cultures grew *Mycobacterium africanum*, which is actually endemic to West Africa and rarely diagnosed in the United States [8]. Our case highlights the need for increased awareness of EPTB and a novel, potentially less invasive, avenue to diagnosis.

## 2. Case Presentation

A 19‐year‐old woman initially presented to our ear, nose, and throat (ENT) outpatient clinic in March 2024 with a one‐month history of a “clogged” sensation in her right ear and intermittent drainage without otalgia. Past medical history was significant for anemia, asthma, and an ear infection in January 2024 that had not responded to several rounds of antibiotic ear drops. She denied a history of ear infection prior to 2024. Otoscopic examination was notable for a right external ear polyp and drainage. She was prescribed ciprofloxacin–dexamethasone ear drops for presumed otitis externa and referred for polyp removal. At follow‐up a week later, she still had ear fullness, but the drainage had improved. Examination continued to show the polyp that was so large it obscured the tympanic membrane. Three weeks later, she underwent noncontrast computed tomography (CT) of the ears, which showed lytic/destructive changes in the right occipital condyle and C1 lateral mass, opacification of the right middle ear cavity and mastoid air cells without bony destructive changes, and opacification of the right external auditory canal (Figure 1). The ear structures on the left side were unremarkable. An audiogram during the same period showed a moderate sloping to profound mixed hearing loss from 250 Hz to 8000 Hz on the right and normal hearing on the left. She was diagnosed with an aggressively erosive cholesteatoma and referred to a tertiary care center for surgical evaluation.

**FIGURE 1:**
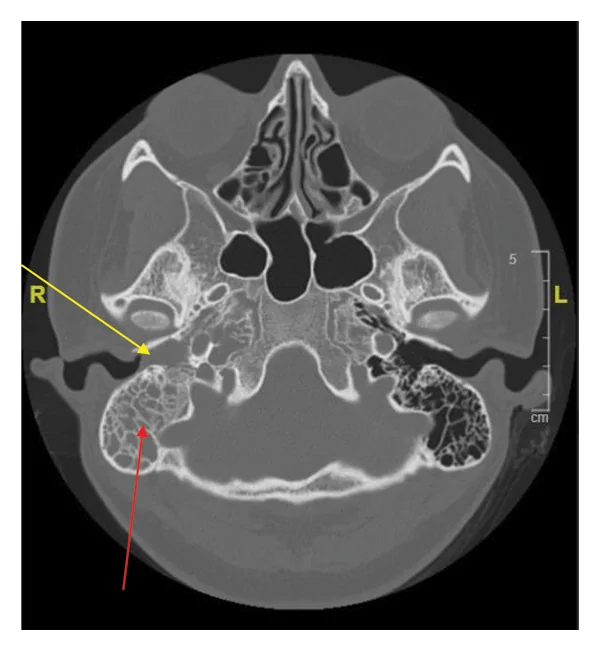
Noncontrast CT of the ears showing opacification of the right middle ear cavity and mastoid air cells (red arrow) without bony destructive changes and opacification of the right external auditory canal (yellow arrow).

In June 2024, she presented to our hospital with a one‐week history of generalized weakness, fatigue, and chills and a one‐day history of neck pain associated with a new lump on her neck. She was tachycardic to 114 beats per minute on evaluation but otherwise afebrile and hemodynamically stable. Laboratory tests were notable for a white blood cell count of 8 k/uL (normal range: 4.5–12.0 K/uL), hemoglobin of 10 g/dL (normal range: 12.0–16.0 g/dL), platelets of 346 K/uL (normal range: 150–400 K/uL), erythrocyte sedimentation rate (ESR) of 67 mm/h (normal range ≤ 20 mm/h), and a C‐reactive protein of 19.5 mm/h (normal range: ≤ 5.0 mg/L). A contrast‐enhanced CT scan of the neck showed erosion of the distal portion of the spinous process of C7 with bony fragmentation. A 3.5‐cm phlegmon surrounded the bony erosion within which multiple abscesses of up to 2 cm had formed. She also had multiple small abscesses along the paraspinal muscles extending superiorly up to the level of the C6 spinous process and inferiorly to the level of the T1 spinous process.

Neurosurgery, infectious diseases (ID), and ENT were consulted, and subsequently the patient underwent magnetic resonance imaging (MRI) of the brain and vertebral spine. MRI of the cervical spine showed osteomyelitis of the left atlanto‐occipital joint and the C6 and C7 spinous processes and multiple abscesses within the C6 and C7 vertebral bodies, the largest of which was 3.9 cm (Figure 2). MRI of the thoracic spine was significant for discitis‐osteomyelitis at T5–T6 with involvement of the T5 vertebral body and the spinous process at C6–C7 (Figure 3). MRI of the brain and internal auditory canal showed enhancement of the right occipital condyle, the right C1 lateral mass, and the right C1 anterior arch extending into the right paraspinal and the right T1–T2 epidural space. There was also a rim‐enhancing 2 × 0.7 × 1.4 cm fluid collection along the posterior aspect of the right atlanto‐occipital joint, a large middle ear effusion on the right, and an enhancing mass in the right middle ear cavity extending into the Eustachian tube. Although prior imaging suggested that the mass in the right ear was a cholesteatoma, this new pattern of enhancement was considered atypical for a cholesteatoma, and broader differentials, including malignancy, were considered.

**FIGURE 2:**
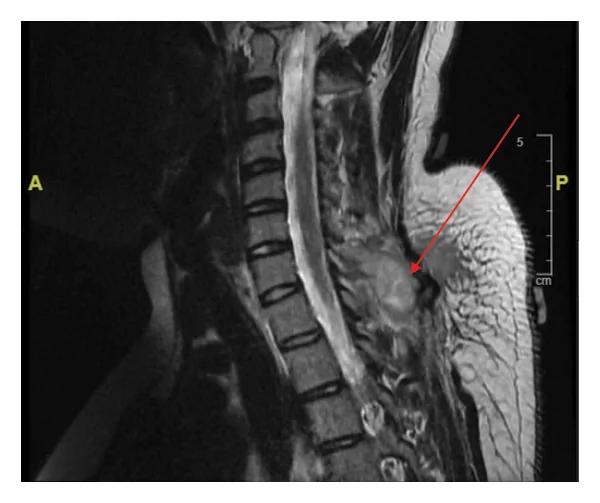
MRI of the cervical spine showing multiple abscess collections (predominantly) involving the left posterior paraspinal soft tissues from C7 to T3 , the largest of these measures 1.5 × 3.9 cm at the level of C7–T1 (red arrow).

**FIGURE 3:**
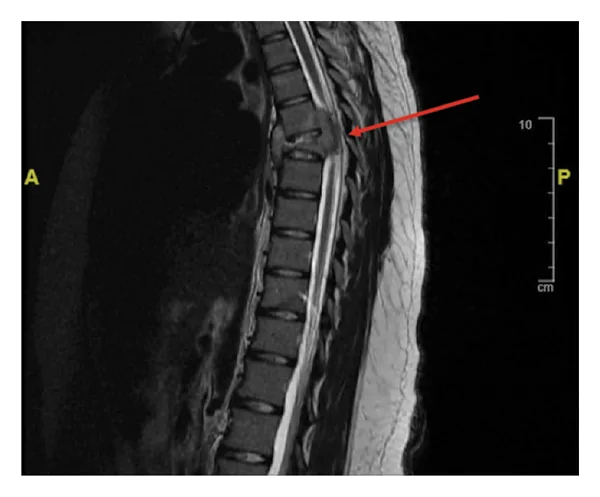
MRI of the thoracic spine with significant posterior bulging of the T6 vertebral body into the spinal canal at the level of T5–T6 causing moderate to severe central canal stenosis and significant mass effect on the spinal cord (red arrow).

Despite the findings on the cervical and thoracic spine, the patient had no neurological symptoms outside of mild neck pain and hearing loss on the right. She received empiric ciprofloxacin and ceftriaxone in the emergency department but was escalated to vancomycin and piperacillin–tazobactam by ID, and a broad evaluation for bacterial, fungal, and mycobacterial etiologies was sent. A screen for human immunodeficiency virus, a 1,3 beta‐D‐glucan test, fungal serologies, and fungal urine and serum antigens were negative. Her CD4 count was 717 cells/μL, and her immunoglobulin levels were normal. On hospital day 4, interventional radiology (IR) performed US‐guided drainage of her neck collection with removal of 40 mL of fluid. Smear and cultures for bacterial, fungal, and mycobacterial organisms and a *Mycobacterium tuberculosis* (MTB) complex PCR of the fluid were ultimately negative.

On hospital day 8, the patient’s QuantiFERON‐TB Gold Plus test came back positive. She also developed fevers to 103 F, prompting escalation of her antibiotic therapy to meropenem. Further history elicited at this time was that the patient was born and raised in the United States and had never left the state of New York. She lived with her grandmother who had emigrated from the Dominican Republic long before the patient was born and had never had TB. The patient herself had never been evaluated for TB before. She was a recent high‐school graduate, but she was not working and had not started college. She was not sexually active, had never been incarcerated, and had never worked in healthcare or with immigrant populations, and all her hobbies were strictly indoors. In short, she did not have any epidemiological risk factors that would explain a positive QuantiFERON‐TB Gold Plus. There was concern for a false‐positive result; however, the TB1 and TB2 antigen values were appropriate at 1.45 IU/mL and 0.98 IU/mL, respectively, and the Mitogen‐NIL was 7.01 IU/mL.

She was placed in isolation to rule out active pulmonary TB as a CT chest performed on hospital day 6 had shown a new left‐sided pleural effusion and enlarged subcarinal lymph nodes. IR deferred a cervical bone biopsy because there was no good window, and neurosurgery held off on an open spine biopsy while awaiting the mycobacterial sputum results. The patient’s mycobacterial smears and MTB complex PCR of the sputum, however, returned negative. To help obtain a tissue diagnosis, ENT took the patient to the operating room (OR) on hospital day 11. Intraoperatively, the patient’s tympanic membrane was noted to be thickened and retracted. There was a pink mass protruding through it, which appeared to be extending from the middle ear into the external auditory canal. They took a biopsy of the part of the mass that had protruded through the tympanic membrane. A frozen section of the biopsy showed extensive lymphocytosis with a giant cell. On hospital day 16, MTB complex PCR from the ear mass came back positive. Pathology showed squamous mucosa with non‐necrotizing granulomatous inflammation and acid‐fast bacilli (AFB). Mycobacterial cultures grew *Mycobacterium africanum*, which was susceptible to all first‐line agents. All MTB complex PCR tests were performed at the Quest Diagnostics laboratory, NYC. Additional rifampin resistance testing was not sent. Initial mycobacterial cultures were all sent to Quest Diagnostics in NYC. They were then forwarded to the Department of Health (DOH) in NYC for mycobacterial susceptibility testing, further identification, and whole‐genome sequencing.

The patient was initiated on rifampin, isoniazid, pyrazinamide, and ethambutol (RIPE), and her other antibiotics were stopped. Liver function testing throughout hospitalization was normal. An ophthalmology examination on hospital day 17 was also normal. She was discharged with follow‐up with ID and the DOH.

At her ID appointment a week after hospitalization, the patient was asymptomatic. All three of her mycobacterial sputum cultures eventually returned negative for *Mycobacterium africanum*. She was subsequently lost to follow‐up with our hospital until November 2025, when she presented to outpatient primary care complaining of muffled hearing in her right ear. She had not been compliant with DOH visits and had stopped RIPE early. Figure 4 summarizes the patient’s case as a timeline of events.

**FIGURE 4:**
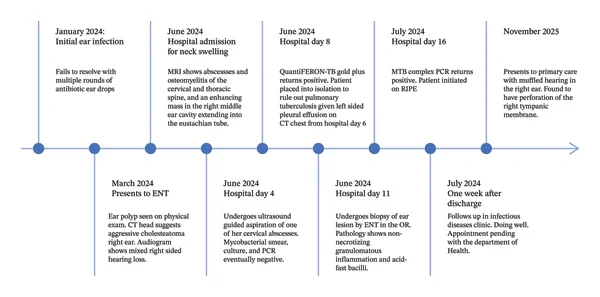
Timeline of events from initial presentation to diagnosis and loss to care.

## 3. Discussion

Diagnosing otic or spinal TB is difficult and often delayed as neither is common. Usually, they are misdiagnosed as more common conditions or missed entirely because initial symptoms can be nonspecific [9]. When the two entities occur together, however, diagnostic accuracy increases as there are few diagnoses that will present with an indolent course and concurrent destruction of the ear and the spine. Aderibige (2006) reported on a patient with otic TB who went undiagnosed for 9 months despite aggressive evaluation for otorrhea, neck pain, and later right hypoglossal nerve palsy until she had an X‐ray of the neck which showed destruction of the cervical bodies of C3–C5 with some narrowing of the intervening disc spaces and a large prevertebral soft tissue collection [6]. Although AFB smears of her otorrhea were negative and mycobacterial cultures were not available, they empirically started the patient on RIPE therapy given the high suspicion for TB in Nigeria. Subsequently, the patient’s otorrhea, neck pain, tympanic membrane perforation, and cervical spine destruction resolved. Yunus (2024) reported on a patient with otic TB and a neck abscess who went undiagnosed for 3 months until he was transferred to a tertiary center where imaging showed a paravertebral abscess, mastoiditis, and osteomyelitis of the bilateral occipital condyles extending to C1 and C2 [7]. He was also placed on RIPE and underwent surgery with full recovery.

Our patient had been experiencing symptoms of otorrhea since January 2024, but because she was born in the United States and had no appreciable exposures, TB was not considered until she presented to the hospital 5 months later with a lump on her neck. Other differentials included bacterial or fungal skull base osteomyelitis with otic involvement, chronic mastoiditis, infection with nontuberculous mycobacteria, malignancy, and sarcoidosis. Bacterial etiologies were ruled out as they did not grow from any of her cultures, and antibiotics did not improve her symptoms. Yeast grew from a bedside ear culture; however, it did not grow from her OR cultures, and all other fungal workup was negative. The non‐necrotizing granulomas on pathology suggested sarcoidosis, but with the AFB seen on stain, a diagnosis of mycobacterial infection fit better. Although necrotizing granulomas are the hallmark, non‐necrotizing granulomas can be seen in TB [10]. We concluded that her initial ear polyp and cholesteatoma were actually a tuberculous middle ear mass that had protruded through the tympanic membrane. Her skull base and spinal findings were all thought to be part of the same process, though we could not determine which came first.

While simultaneous presentation with otic and spinal processes raises the index of suspicion for EPTB, making the diagnosis remains challenging. Unlike in pulmonary TB, EPTB is often paucibacillary, and initial smears and even PCRs can be negative [5]. Tissue diagnosis is the gold standard, but choosing where to obtain the tissue can often be complicated. Our initial approach was to be as noninvasive as possible. Thus, the patient had an IR‐guided aspiration of the fluid from her neck with collection of mycobacterial cultures and an MTB complex PCR, but these were negative. We suspect this was because of a low bacillary burden. We also collected multiple mycobacterial sputum smears and cultures, an MTB complex PCR on her sputum, and a mycobacterial blood culture, in the hopes that if this was disseminated TB; other sites might also be positive. All of these were also negative. For quite some time, our only clue that this could be a mycobacterial infection was that her QuantiFERON‐TB Gold Plus was positive, which at least suggested exposure. Without the biopsy from the ear, we would have likely never been able to substantiate our diagnosis enough to start RIPE without an open biopsy of her spine as there was no good window for an IR‐guided biopsy. Ear biopsy is a fairly common diagnostic method for otic TB and, according to a systematic review by Pai et al., is also reliable. Of the 67 cases they reviewed, 53 were diagnosed by histopathology [11]. Sensitivity improves when paired with MTB complex PCR [12]. Our case suggests that it can also be used to support a diagnosis of Pott’s disease with the presumption that whatever is happening in the ear is also happening in the spine. This can help avoid invasive neurosurgical procedures as Pott’s disease, in the absence of neurological compromise, does not require surgical intervention for treatment [13].

Our patient was planned for 9–12 months of RIPE with repeat imaging to determine her ultimate duration. Unfortunately, she was poorly compliant with DOH visits, deferred directly observed therapy from the DOH, and never followed up again at our ID office for repeat imaging or audiology screening. When she presented to primary care in November 2025 with abnormal hearing, she did not have fevers, chills, night sweats, or back pain. Examination noted a 2‐mm perforation of her right eardrum. She was estimated to have only taken 2–3 months of her planned course of RIPE. Outreach was attempted to bring her back into care with the DOH and ID; however, she missed all subsequent appointments to restart treatment. At the time of this paper’s publication, she is alive; however, her clinical status is unknown.

According to the Centers for Disease Control and Prevention, the incidence of TB in the United States was 3.0 cases per 100,000 in 2024 [14]. In New York City, however, the case rate is much higher at 9.5 per 100,000 population and is one of the highest in the United States. Most cases are diagnosed in the Bronx and Queens, and the majority of cases are in foreign‐born individuals [15]. Our patient did live in the Bronx but did not have any other conventional exposures to TB. Even more interesting, she grew *Mycobacterium africanum*, which is endemic to West Africa and incredibly rare in the United States. One review estimated the incidence at only 0.4% of TB cases diagnosed between 2004 and 2013 [8]. When diagnosed, it is usually among individuals who are of West African descent or are non‐Hispanic Black individuals, neither of which described our patient. Our patient’s case on its own cannot prove ongoing transmission of TB in NYC, but it does lend support to keep it on the differential even among those without obvious risk factors.

## 4. Conclusion

Simultaneous spinal and otic TB cases are rare and require a high index of suspicion to diagnose. Biopsy of a lesion in the ear could be a less invasive way to support a diagnosis of concurrent spinal TB and avoids invasive neurosurgical procedures. Increases in the incidence of TB in NYC should raise suspicion for the disease in individuals with no known risk factors.

## Author Contributions

Sabirah N. Kasule: conceptualization, writing–original draft, investigation, and writing–review and editing; George S. Zacharia: conceptualization, writing–original draft, and investigation; Abelard C. Desrosiers: writing–original draft; Shalini Penikilapate, Marcos A. Molina, and Srinivasan Krishna: investigation, review and editing; Cosmina B. Zeana: supervision and writing–review and editing.

## Funding

No funding was required for this case report.

## Consent

Informed consent was obtained from the subject in this case report for publication.

## Conflicts of Interest

The authors declare no conflicts of interest.

## Supporting Information

Additional supporting information can be found online in the Supporting Information section.

## Supporting information


**Supporting Information** CARE‐checklist‐English‐2013.

## Data Availability

Data sharing is not applicable to this article as no datasets were generated or analyzed during the current study.
