# Supplementary material for: Concurrent Middle Ear and Vertebral Tuberculosis Secondary to Mycobacterium Africanum in an Immunocompetent Young Adult in a Low‐Incidence Setting: Diagnostic Value of Middle Ear Biopsy
Source: Case Rep Infect Dis. 2026 Oct 16;2026:5812867. doi: 10.1155/crdi/5812867 (PMC13674006; doi:10.1155/crdi/5812867)
Supplement: Supplementary file 1 [file CRDI-2026-5812867-s001.pdf]

CARE
case report guidelines

CARE Checklist of information to include when writing a case report

(cc) BY-NC-ND

Topic	Item	Checklist item description	Reported on Line
**Title**	**1**	The diagnosis or intervention of primary focus followed by the words "case report"	Title Page
**Key Words**	**2**	2 to 5 key words that identify diagnoses or interventions in this case report, including "case report"	Page 2
**Abstract (no references)**	**3a**	Introduction: What is unique about this case and what does it add to the scientific literature?	1-2
	**3b**	Main symptoms and/or important clinical findings	5-8
	**3c**	The main diagnoses, therapeutic interventions, and outcomes	9-12
	**3d**	Conclusion—What is the main "take-away" lesson(s) from this case?	
**Introduction**	**4**	One or two paragraphs summarizing why this case is unique (**may include references**)	20-25
**Patient Information**	**5a**	De-identified patient specific information.	35-37
	**5b**	Primary concerns and symptoms of the patient.	33-35
	**5c**	Medical, family, and psycho-social history including relevant genetic information	35-37
	**5d**	Relevant past interventions with outcomes	36
**Clinical Findings**	**6**	Describe significant physical examination (PE) and important clinical findings.	37, 48-49
**Timeline**	**7**	Historical and current information from this episode of care organized as a timeline	page 14
**Diagnostic Assessment**	**8a**	Diagnostic testing (such as PE, laboratory testing, imaging, surveys).	48-71
	**8b**	Diagnostic challenges (such as access to testing, financial, or cultural)	N/A
	**8c**	Diagnosis (including other diagnoses considered)	105
	**8d**	Prognosis (such as staging in oncology) where applicable	N/A
**Therapeutic Intervention**	**9a**	Types of therapeutic intervention (such as pharmacologic, surgical, preventive, self-care)	111
	**9b**	Administration of therapeutic intervention (such as dosage, strength, duration)	111
	**9c**	Changes in therapeutic intervention (with rationale)	83
**Follow-up and Outcomes**	**10a**	Clinician and patient-assessed outcomes (if available)	115-120, 169-176
	**10b**	Important follow-up diagnostic and other test results	111
	**10c**	Intervention adherence and tolerability (How was this assessed?)	169
	**10d**	Adverse and unanticipated events	115-120, 169-176
**Discussion**	**11a**	A scientific discussion of the strengths AND limitations associated with this case report	121-187
	**11b**	Discussion of the relevant medical literature **with references**.	121 - 136
	**11c**	The scientific rationale for any conclusions (including assessment of possible causes)	146-149
	**11d**	The primary "take-away" lessons of this case report (without references) in a one paragraph conclusion	190-194
**Patient Perspective**	**12**	The patient should share their perspective in one to two paragraphs on the treatment(s) they received	N/A
**Informed Consent**	**13**	Did the patient give informed consent? Please provide if requested	**Yes** ☒ **No** ☐
